# Supplementary material for: Postmortem imaging reveals patterns of medial temporal lobe vulnerability to tau pathology in Alzheimer’s disease
Source: Nat Commun. 2024 Jun 5;15:4803. doi: 10.1038/s41467-024-49205-0 (PMC11153494; doi:10.1038/s41467-024-49205-0)
Supplement: Supplementary file 1 — Supplementary Information [file 41467_2024_49205_MOESM1_ESM.pdf]

Supplementary Information

1 Supplementary Methods

1.1 Ex vivo MRI Acquisition and Processing

Following fixation, temporal lobe specimens were scanned in a cylindrical container containing an MRI-neutral industrial lubricant. The specimens were imaged with a custom-made 70 mm coil, using an acrylic holder with a 49.8 mm inner diameter and a long (80 mm) z-field. Prior to scanning, special precaution was taken to eliminate air bubbles trapped in both the brain and container by gently agitating the specimen by hand while submerged in the MRI-neutral lubricant. MRI scans were obtained using a multi-slice spin echo sequence. Sequence parameters varied slightly between specimens, with typical values being a repetition time of 9330 ms and an echo time of 23 ms. Following image acquisition, the images were corrected for bias field non-uniformity using the N4ITK algorithm [10] and normalized to a common intensity range by clipping the intensities below the 0.1 and above the 99.9 percentile, and scaling the intensity range to [0,1000]. Due to an error in the scanner gradient calibration, as part of the post-processing, all the scans had to be linearly scaled (6% in x, 3% in y, 11% in z) to correct for differences between the scanner coordinate frame and physical coordinate frame. These linear scaling factors were derived using a 3D printed phantom [1]. In Ravikumar et al. 2021, we found that the applied linear scaling factors have no effect on our cortical thickness measurements and the resulting pathology-structure correlation analyses when compared to analyses using native MRI space measurements [8].

1.2 Automated MTL and SRLM segmentation in ex vivo MRI

Segmentations of the medial temporal lobe (MTL) cortex and stratum radiatum lacunosum moleculare (SRLM), the thin, hypo-intense layer within the hippocampus, were used to guide groupwise registration for atlas construction. In our previous work, we adopted a contour and intensity-guided interpolation approach for MTL segmentation, and a deep-learning based approach to initialize SRLM segmentation [8]. While these methods reduced the time it took to obtain complete 3D segmentations of the MTL and SRLM, more recently, we have developed a more automated pipeline based on a single volumetric convolutional neural network (CNN) which performs both MTL and SRLM segmentation. Our segmentation pipeline is based on a modified implementation of the nnU-Net framework [4] that has been trained using a custom Laplacian-based loss function that we developed to improve the detection of sulci in the cortex [9]. More details are provided in the following sections.

1.2.1 Ground truth MTL segmentations

To train the CNN, 3D segmentations of the MTL cortex were generated for ex vivo MRI scans of 27 specimens using the inter-slice interpolation technique used in previous work [7, 8]. According to the protocol we follow, the segmented region within the atlas includes the hippocampus, entorhinal cortex, perirhinal cortex (PRC) (the medial bank of the occipitotemporal sulcus served as the anterior lateral border), and parahippocampal cortex (PHC) (the fundus of the collateral sulcus served as the posterior lateral border). Each of the 27 MTL segmentations was manually edited in narrow and bridged sulci to ensure that the resulting reconstruction was geometrically accurate. Additionally, in a small region surrounding the MTL, the white matter and background voxels were semi-automatically labelled using a combination of intensity-based thresholding and morphological operations.

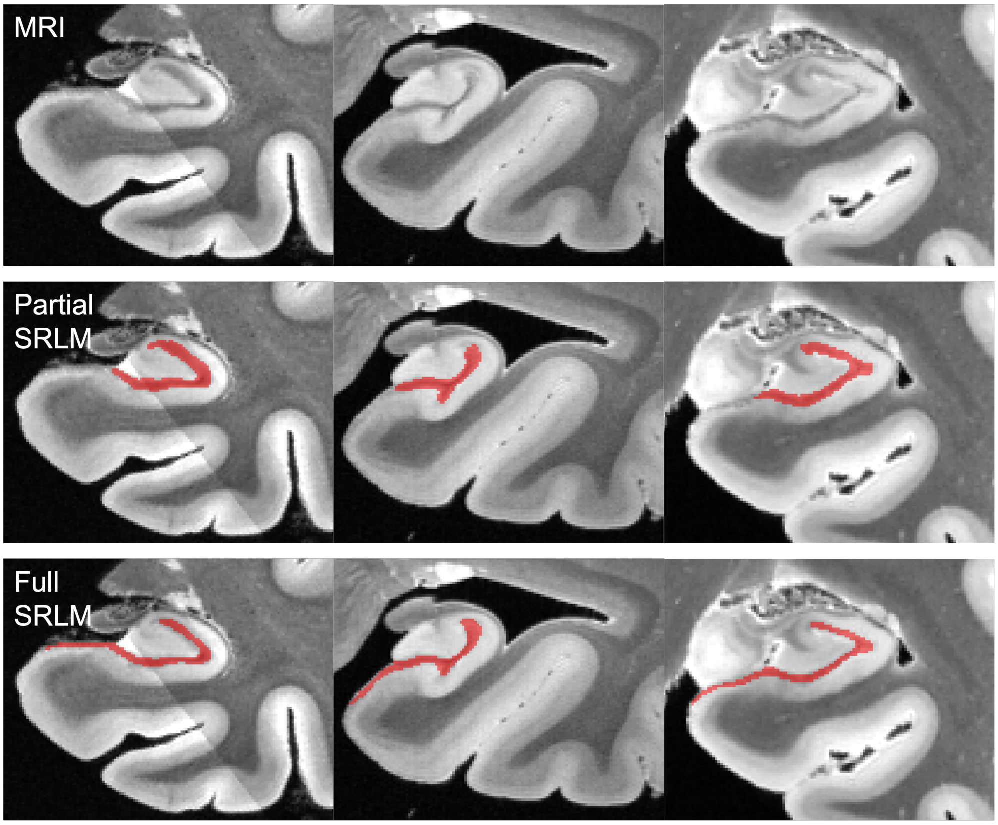

Supplementary Figure 1: **Cross-sectional view of the medial temporal lobe (MTL) in three specimens comparing the extents of stratum radiatum lacunosum moleculare (SRLM) label included the previous and current atlas.** The full SRLM label, includes the perforant pathway (PP) and extends over the full subiculum, whereas in the previously developed atlas, the SRLM only covered the more medial portion of the subiculum.

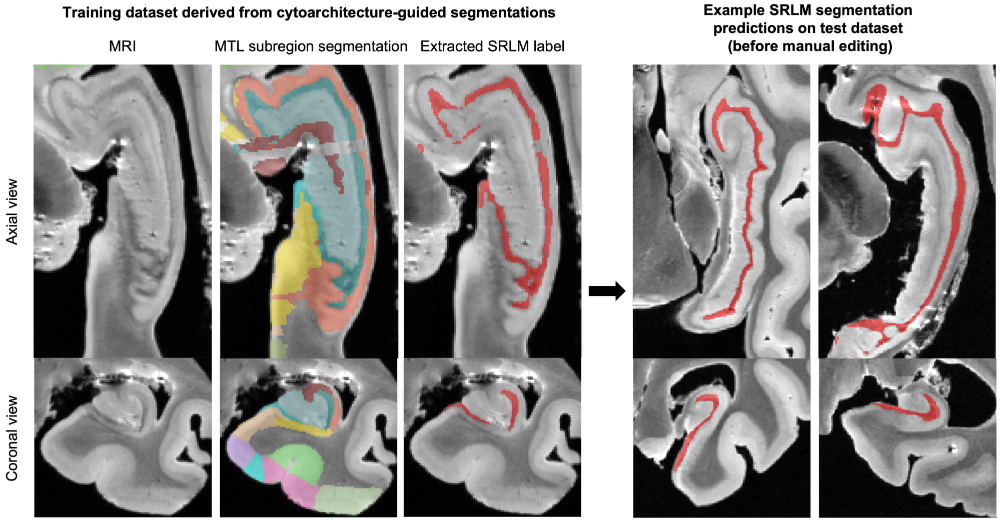

Supplementary Figure 2: **Cross-sectional view in an example specimen, showing the histology-guided subregion segmentation and the extracted stratum radiatum lacunosum moleculare (SRLM) label used to train the segmentation network.** Despite missing regions in the ground truth SRLM label due to gaps between tissue blocks, and the size of the training dataset (n = 11), the network learns to predict the entire SRLM label.

1.2.2 Ground truth SRLM segmentations

The ground truth segmentations also contain a separate label for the SRLM. For constructing the atlas presented in [8], segmentations of the SRLM in this dataset were initialized using a CNN trained using existing manual segmentations from Adler et al. [1] which only included the SRLM of the CA subfields and the medial portion of the molecular layer of subiculum. Since the molecular layer in the atlas only partially extended over the subiculum, this limited our ability to accurately measure thickness in the full subiculum region. As shown in Supplementary Figure 3, in the current work, we extend the SRLM segmentations in our dataset to include the perforant pathway, which refers to the hypo-intense layer extending over the entire length of the subiculum, presubiculum and parasubiculum. This extension was done by leveraging a dataset of histology guided MTL subregion segmentations manually completed in MRI scans from 11 specimens, that include labels for both the SRLM and perforant pathway. Using this smaller dataset, we adopted a two-stage approach to automatically generate extended segmentations of the SRLM in each of our ex vivo MRI scans.

First, we extracted the SRLM and perforant pathway labels from the 11 histology-based segmentations to serve as training data, and then trained a segmentation network using the widely successful nnU-Net framework [4] (Supplementary Figure 4). The 5-fold cross validated Dice score coefficient (DSC) of the predicted SRLM segmentations is 75.3%. We note that since the training segmentations were derived from histology, small gaps in the ground truth segmentation sometimes exist between tissue blocks, thus lowering the DSC reported by the network. To further improve the accuracy of the predicted segmentations, this initial model was applied to the larger dataset of 27 MRI scans for which manual MTL segmentations were already completed (section 1.2.1) to update the SRLM segmentations and generate more training data. The predicted SRLM segmentations for these cases were manually edited for minor errors. The corrected SRLM segmentations were then incorporated into the ground truth multi-label segmentations described in section 1.2.1, containing the MTL gray matter and surrounding white matter and background labels. This dataset was then used to train the custom-developed CNN framework described in the next section.

1.2.3 Supervised deep learning-based cortical segmentation.

In recent work, we have developed an automated pipeline based on a single volumetric deep segmentation network which performs both MTL and SRLM segmentation. In addition to performing voxel-wise labelling of the MTL and SRLM in each scan, the developed network aims to generate cortical segmentations that are geometrically accurate by enforcing the presence of sulci, particularly in regions where the sulci are buried between tightly folded cortex and not easily visible. In brief, the developed approach builds upon the nn-Unet framework by incorporating a differentiable numerical solver for Laplace's equation after the final layer of the network. During training, the numerical solver computes the Laplacian mapping between the predicted white matter and pial surfaces for a given input image and, using a custom loss function, compares the predicted tissue segmentations and corresponding Laplacian field maps with the equivalent ground truth images, thus penalizing self-intersections in the predicted segmentations. Our results show that when compared to the baseline nn-Unet framework trained without Laplacian constraints, our method is able to better detect buried sulci and reconstruct the intrinsic, layered geometry of the cortex. More details of the network architecture are provided in [9].

The developed network requires the Laplacian field maps corresponding to the ground truth segmentations to train the model. To solve Laplace's equation within the ground truth cortical segmentation, we used the iterative finite-differences approach, as implemented in publicly available MATLAB code provided by [2]. After computing the ground truth Laplacian maps, we trained the segmentation network using patches of size 96 x 96 x 96 extracted from the ex vivo MRI scans, ground truth MTL segmentations and corresponding Laplacian solutions for 27 specimens. The network was trained to predict four labels: gray matter cortex within the MTL, SRLM, white matter and background and the final cross-validated accuracy of the predicted MTL and SRLM labels is 94.5% and 87% respectively.

1.3 Computation of regional MTL thickness

In our previous work [8], we estimated regional thickness of the MTL cortex and SRLM by warping the template segmentations to the native MRI space of each specimen and performing Voronoi skeletonization [6]. Thickness measurements for each specimen were then brought back into template space and mesh-based statistical analysis was performed along the boundary surface of the MTL and SRLM templates. In the current work, we directly estimate regional thickness by skeletonizing each specimen's distortion-corrected segmentations and map the thickness measurements onto the skeleton of the template to perform mesh-based statistical analysis. First, surface meshes were extracted from the distortion-corrected MTL and SRLM segmentations of each specimen using the marching cubes algorithm [5]. For each surface mesh, the Taubin smoothing algorithm was used to remove sharp edges before Voronoi skeletonization and Delaunay tetrahedralization were applied to extract the pruned skeleton and boundary tetrahedral respectively. The skeletonization step also outputs a corresponding "thickness image" which specifies the thickness at each voxel within the MTL and SRLM segmentations. Thickness was estimated along each tetrahedral mesh as the distance from the skeleton vertices to the boundary generator points. To bring the thickness measurements into the space of the template, the skeletons of the MTL and SRLM templates were extracted and warped into the space of each specimen using the transformations generated by the groupwise registration pipeline. Thickness measurements for each specimen were then mapped onto the template's skeleton by sampling the closest point on a specimen's boundary tetrahedral for each vertex along the skeleton. In regions where the vertex of the warped skeleton falls outside of the boundary tetrahedral, the vertex is assigned the value of the closest boundary cell that is less than 0.6mm away, otherwise it is assigned a missing value (NaN). Additionally, at certain vertices in some specimens, thickness measurements were assigned a missing value in regions where the MRI scan is affected by image artifacts due to missing tissue and tearing. Furthermore, to minimize unreliable thickness measurements in regions of poor registration between a specimen and template, we excluded thickness measurements at vertices where the magnitude of the deformation was in the top 0.1% of the warp image. This resulted in a variable number of observations at each vertex and was accounted for in the statistical analysis. For the region of interest (ROI) analyses, a summary thickness measure was generated for each MTL subregion label in each specimen using the thickness images output by the skeletonization step by computing the mean thickness value across all voxels in that anatomical ROI, while

also taking into account missing values due to image artifacts and registration errors.

2 Supplementary Results and Figures

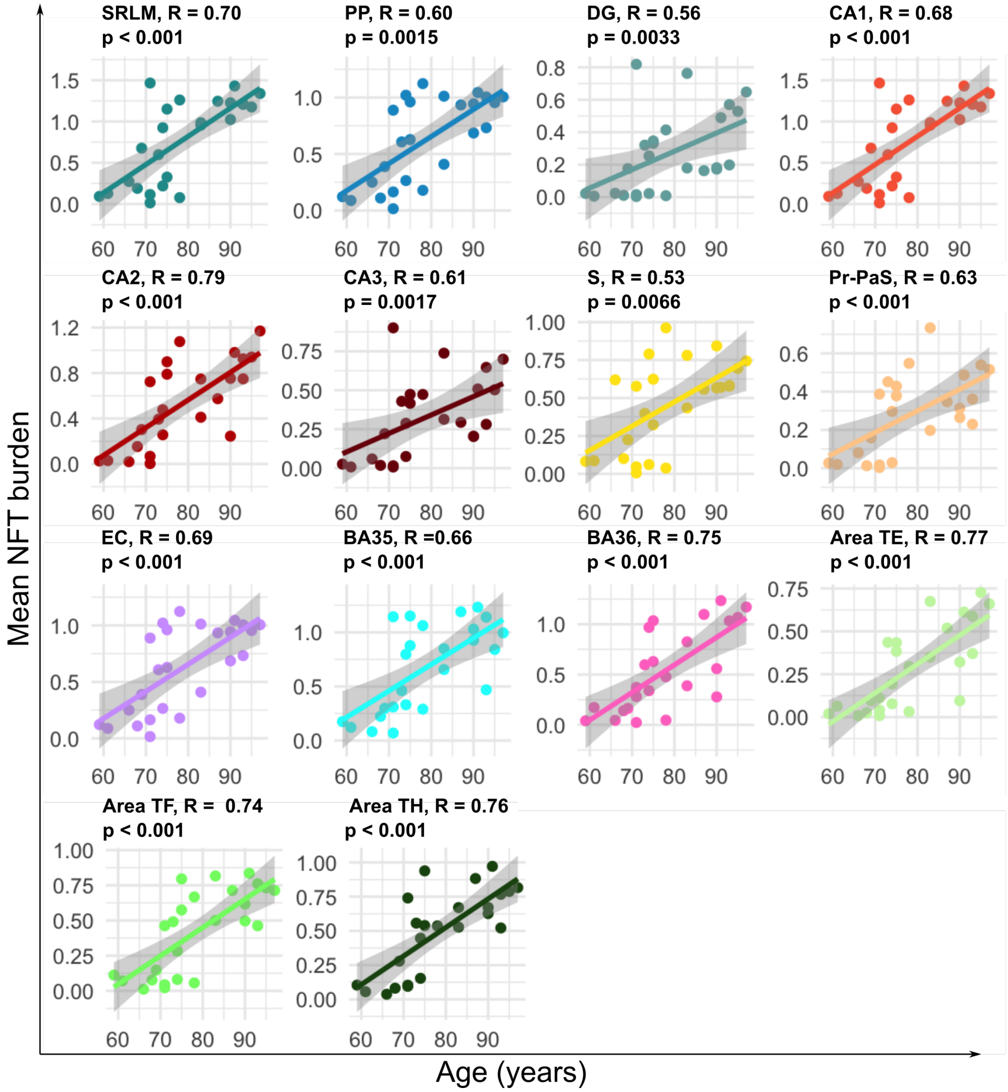

Supplementary Figure 3: **Scatter plots illustrating the relationship between age and mean NFT burden computed within each of the 14 MTL subregions. Each plot also includes the corresponding Spearman's rank correlation calculated between age and mean NFT burden.** The two exceptions are SRLM and PP since these ROIs do not directly accumulate NFT pathology. For SRLM and PP, age is correlated with mean NFT burden in CA1 and the entorhinal cortex region respectively. Significant positive associations (two-sided, $p < 0.05$) between age and NFT burden are observed across all subregions. Sample sizes are provided in Supplementary Table 2. (S = subiculum; PrS-PaS: Pre/Parasubiculum; SRLM = stratum radiatum lacunosum moleculare; PP: perforant pathway; CA = cornu ammonis; DG = dentate gyrus; ERC = entorhinal cortex; BA = Brodmann Area)

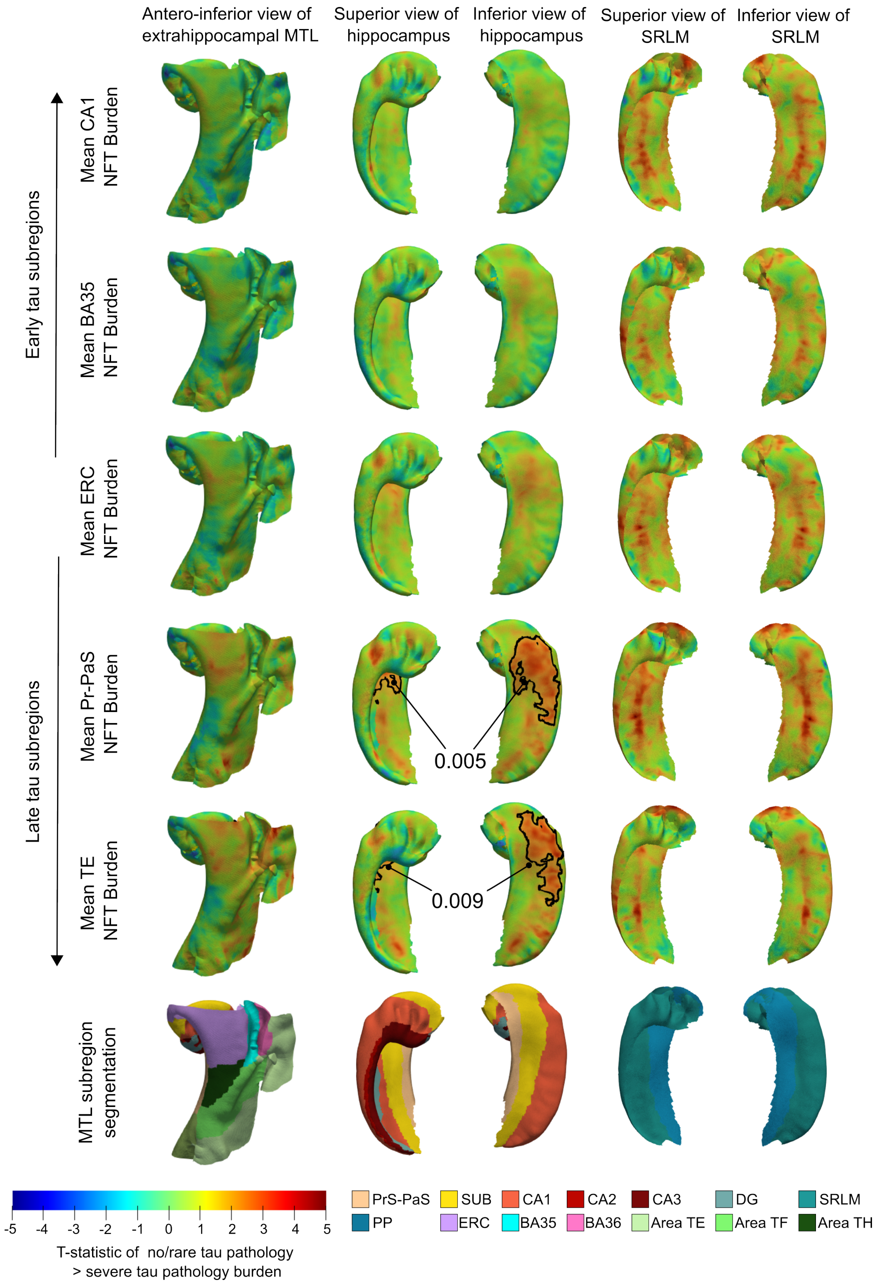

Supplementary Figure 4: **Association between pointwise medial temporal lobe thickness and ipsilateral, quantitative NFT burden measures, with age and sex included as additional co-variates in the model.** The t-statistic maps show the correlation between pointwise cortical thickness and the mean NFT burden computed within different anatomical subregions ($n = 25$). The NFT burden measures are computed based on the 3-D quantitative NFT density maps. The analyses are arranged in the order in which the anatomical subregions used is affected during the AD process, from early to late, based on the results shown in Fig. 2C. Clusters were defined based on an empirical threshold (uncorrected $p < 0.01$) and permutation testing with the Freedman & Lane method (1000 iterations) was used to assign each potential cluster a one-sided, corrected p-value. To account for multiple comparisons, the analysis uses cluster-level family-wise error rate correction. The clusters outlined in black indicate regions where a significant correlation was observed after correction for multiple hypothesis testing (corrected $p < 0.05$). (S = subiculum; PrS-PaS: Pre/Parasubiculum; SRLM = stratum radiatum lacunosum moleculare; PP: perforant pathway; CA = cornu ammonis; DG = dentate gyrus; ERC = entorhinal cortex; BA = Brodmann area)

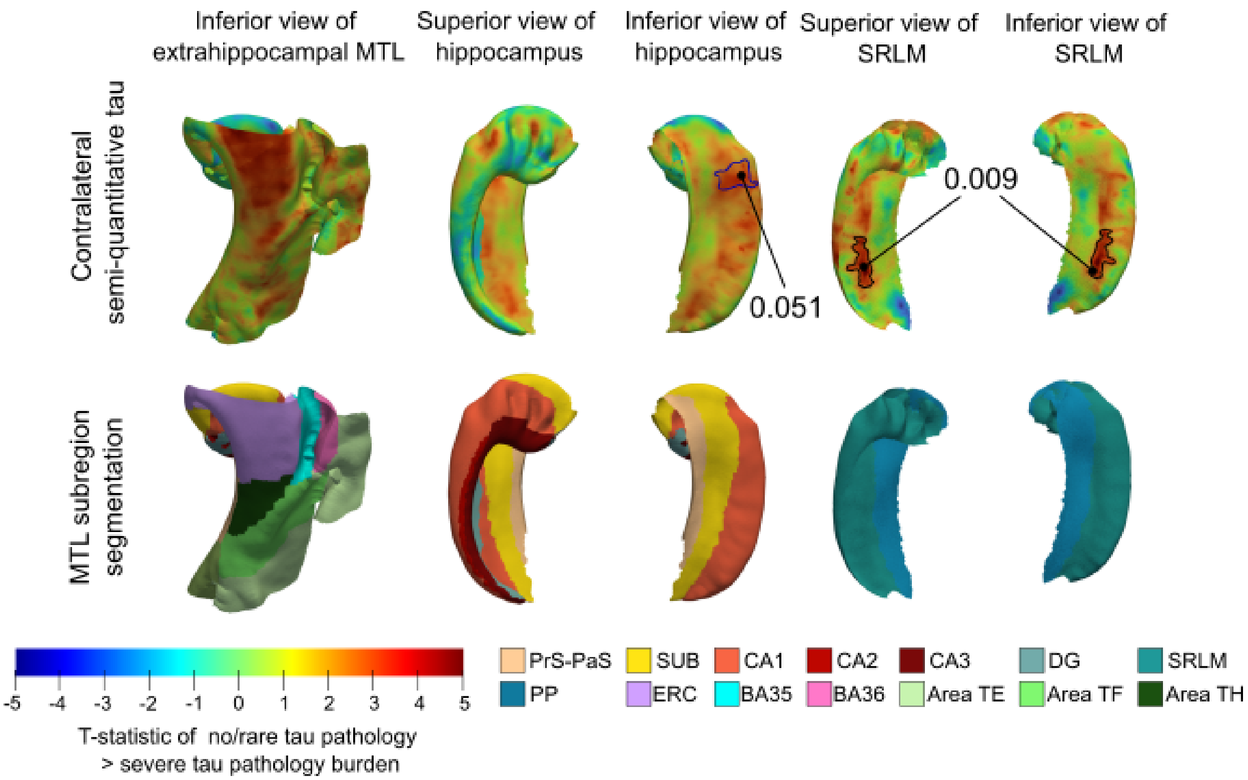

Supplementary Figure 5: **T-statistic maps showing the correlation between pointwise cortical thickness and semi-quantitative tau ratings based on tissue samples obtained from the MTL contralateral to the thickness measures (n = 25).** This analysis uses the same set of cases used in Figure 5 (Section 2.3). No covariates were included in this model. Clusters were defined based on an empirical threshold (uncorrected $p < 0.01$) and permutation testing with the Freedman & Lane method (1000 iterations) was used to assign each potential cluster a one-sided, corrected p-value. To account for multiple comparisons, the analysis uses cluster-level family-wise error rate correction. The clusters outlined in black indicate regions where a significant correlation was observed after correction for multiple hypothesis testing (corrected $p < 0.05$) and the cluster outlined in blue indicates a trend-level correlation (corrected $p < 0.1$). (S = subiculum; PrS-PaS: Pre/Parasubculum; SRLM = stratum radiatum lacunosum moleculare; PP: perforant pathway; CA = cornu ammonis; DG = dentate gyrus; ERC = entorhinal cortex; BA = Brodmann area)

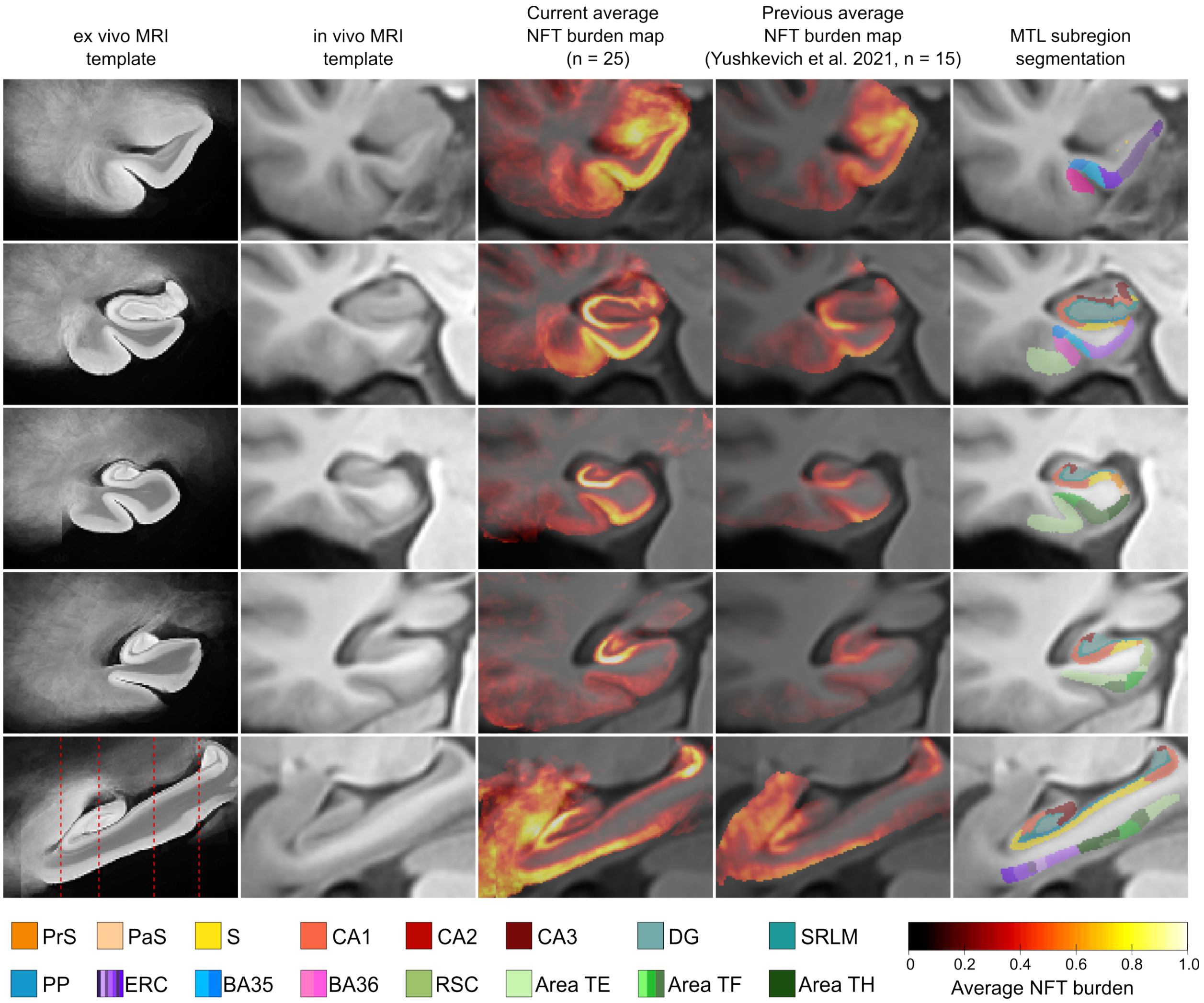

Supplementary Figure 6: **Side-by-side comparison of the average NFT burden map computed in the current work (n = 25) and the average NFT burden map computed in Yushkevich et al. [11] (n = 15), both visualized in the space of an in vivo human brain MRI template.** The ex vivo atlas was mapped to the in vivo atlas using highly smoothed deformable registration applied to simplified MTL segmentations defined in each space. (S = subiculum; PrS-PaS: Pre/Parasubculum; SRLM = stratum radiatum lacunosum moleculare; PP: perforant pathway; CA = cornu ammonis; DG = dentate gyrus; ERC = entorhinal cortex; BA = Brodmann area)

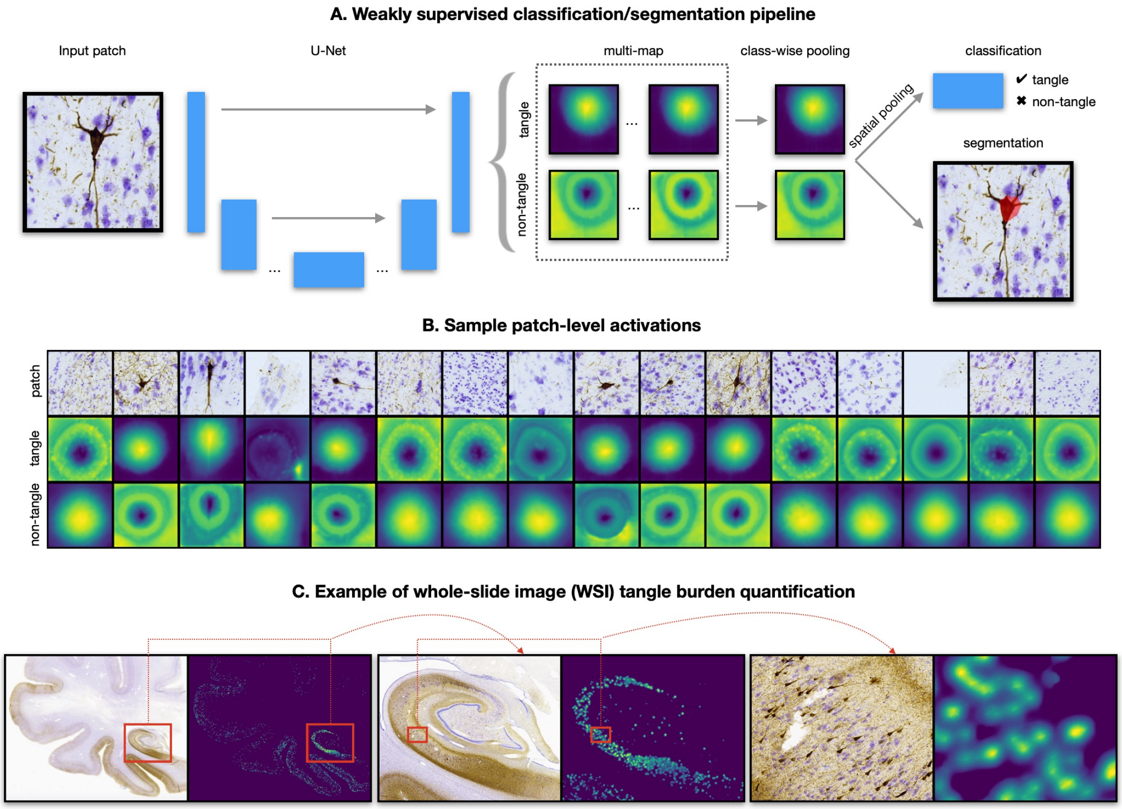

Supplementary Figure 7: **Weakly supervised segmentation pipeline used to derive 3D tau tangle burden maps. A)** The convolutional neural network is based on the WildCat algorithm (Durand et al., 2017 [3]). Input patches are passed to a U-Net, which generates a set of activation maps, called "multi-maps", for each class label (tangle, non-tangle). The use of multi-maps, proposed by Durand et al., is to account for the possibility of different visual presentations of members of the same class; however, in our experiment, the multi-maps are nearly identical to each other. Multi-maps for each class are averaged ("class pooling" of Durand et al.) to construct a per-class activation map. These activation maps are reduced to a small number of values ("spatial pooling" of Durand et al.) that are passed to the classifier. During training, the cross-entropy loss between the output of the classifier and true patch labels is used to update the weights of the U-net. The activation maps can also be thresholded to yield a rough segmentation of the image region driving the classification decision, i.e., individual tangles. **B)** Examples of activation maps (output of the class pooling layer) generated at inference time by a trained model for sixteen sample patches from specimens not seen by the model during training. The activation map generally agrees with the location of the tangles in patches that contain them. **C)** Example of the same trained model applied to a whole-slide image (WSI), shown at different zoom levels. The heat map corresponds to the difference between the tangle and non-tangle activation maps, thresholded at zero. This map is used as the measure of tangle burden in this paper. Prior work (Yushkevich et al., 2021, Brain) validated this tangle burden measure by comparing its value to manual tangle counts and an expert's semi-quantitative rating of tangle severity in regions of interest sampled from WSI not seen by the classifier during training.

Supplementary Table 1: Extended demographics. Diagnostic information is included for the 47 subjects with an "AD continuum" diagnosis and no confounding non-AD tau or frontotemporal lobar degeneration-TDP43 pathology (FTLD-TDP-43) that were included in the pathology-structure correlation analyses. This excludes CNDR01-CNDR07 i.e. cases with Pick's disease (PD), Argyrophilic grain disease (AGD), progressive supranuclear palsy (PSP), corticobasal degeneration (CBD) and FTLD-TDP43. (ADNC: Alzheimer's Disease Neuropathologic Change; LBD: Lewy Body Disease; LATE: Limbic Age-related TDP-43 Encephalopathy; PART: Primary Age-Related Tauopathy; CVD: Cerebrovascular disease; PMI: Post-mortem interval)

**Donor ID**	**3D NFT Burden Map?**	**MTL Subregion Labels?**	**Age**	**Sex**	**PMI (hours)**	**APOE Status**	**Global Pathology Score (NIA/AA Criteria)**			**Neuropathological Diagnosis**		**Contralateral MTL Average Pathology Burden**			
							**A**	**B**	**C**	**Primary**	**Secondary**	**Tau**	**TDP-43**	**a-synuclein**	**AB**
**CNDR01**		Y													
**CNDR02**		Y													
**CNDR03**		Y													
**CNDR04**		Y													
**CNDR05**		Y													
**CNDR06**		Y													
**CNDR07**		Y													
**CNDR08**			74	M	25	E3/E3	3	2	2	LBD	High ADNC	2	0	1.67	2
**CNDR09**			86	F	21	E2/E3	3	3	3	High ADNC	Possible LBD, CVD	1.83	0	0	2.33
**CNDR10**			73	F	15	E3/E3	2	2	1	LBD	Intermediate ADNC	2.33	0	0.67	0.17
**CNDR11**	Y		75	F	8	E3/E4	2	2	2	CVD	Intermediate ADNC	2	0	0	1
**CNDR12**	Y	Y	75	M	9	E3/E4	3	3	3	High ADNC		2.67	0	0	1.33
**CNDR13**	Y		73	F	22	E3/E3	3	3	3	High ADNC		2.33	0.67	0	2.33
**CNDR14**		Y	73	F	19	E3/E3	1	1	1	Multiple system atrophy	CVD, Low ADNC		0	1.67	0
**CNDR15**			75	M	19	E4/E4	3	3	3	High ADNC	Possible LBD, CVD	2.33	0	0.17	2
**CNDR16**	Y	Y	83	M	9	E3/E3	3	2	3	LBD	Intermediate ADNC	2.17	0	1	2
**CNDR17**	Y		69	M	22	E4/E4	3	3	3	LBD	High ADNC	1.67	0	2.33	2.33
**CNDR18**	Y	Y	71	M	18	E4/E4	3	2	1	LBD	Intermediate ADNC	2.67	0	2.17	2.33
**CNDR19**	Y	Y	87	M	14.5	E3/E4	3	2	0	Intermediate ADNC		2.33	0	0	1.67

Supplementary Table 1: Extended demographics. Diagnostic information is included for the 47 subjects with an "AD continuum" diagnosis and no confounding non-AD tau or frontotemporal lobar degeneration-TDP43 pathology (FTLD-TDP-43) that were included in the pathology-structure correlation analyses. This excludes CNDR01-CNDR07 i.e. cases with Pick's disease (PD), Argyrophilic grain disease (AGD), progressive supranuclear palsy (PSP), corticobasal degeneration (CBD) and FTLD-TDP43. (ADNC: Alzheimer's Disease Neuropathologic Change; LBD: Lewy Body Disease; LATE: Limbic Age-related TDP-43 Encephalopathy; PART: Primary Age-Related Tauopathy; CVD: Cerebrovascular disease; PMI: Post-mortem interval)

**Donor ID**	**3D NFT Burden Map?**	**MTL Subregion Labels?**	**Age**	**Sex**	**PMI (hours)**	**APOE Status**	**Global Pathology Score (NIA/AA Criteria)**			**Neuropathological Diagnosis**		**Contralateral MTL Average Pathology Burden**			
							**A**	**B**	**C**	**Primary**	**Secondary**	**Tau**	**TDP-43**	**a-synuclein**	**AB**
**CNDR20**			93	M	16	E3/E3	1	1	0	LATE	Hippocampal Sclerosis, Low ADNC	1	2.33	0	0
**CNDR21**			88	F	16	E3/E3	2	2	2	Intermediate ADNC		1.17	0.33	0	1.33
**CNDR22**	Y		97	M	18	E3/E3	2	2	1	Intermediate ADNC		1.67	0	0	1.17
**CNDR23**	Y		83	M	4	E3/E4	3	3	3	High ADNC		3	0.17	0	2.67
**CNDR24**			78	F	12	E3/E3	3	3	3	High ADNC		2.33	0	0	2.33
**CNDR25**			76	F	45	E3/E4	3	3	3	High ADNC		2.17	0	0	2.33
**CNDR26**			75	M	8	E3/E4	2	2	3	Intermediate ADNC		2	0	0	1.83
**CNDR27**			74	M	8	E3/E4	3	3	3	High ADNC	LBD, LATE	2.67	1	0.5	2.67
**CNDR28**			75	M	18.5	E3/E3	3	3	3	High ADNC	LBD	2.67	0	1.33	2.33
**CNDR29**			89	F	4	E3/E3	0	2	0	LATE	PART, LBD	2.33	1.67	0	0
**CNDR30**			74	M	30.5	E3/E3	0	1	0	LBD	CVD, PART	0.33	0	0	0
**CNDR31**			78	M	11.5	E3/E3	1	1	0	LBD	Intermediate ADNC	1.17	0	1	0
**CNDR32**			87	M	19	E3/E3	3	3	3	High ADNC		2.67	0	0	2.33
**CNDR33**			84	F	24	E3/E3	2	1	0	Low ADNC		0.67	0	0	1
**CNDR34**			99	M	41	E2/E3	1	1	2	Low ADNC		1.5	0	0	0.17
**HNL01**			81	M	16		2	0	2	Low ADNC		0.5	0	0	1
**HNL02**			68	M	11		0	0	0	Unremarkable Adult Brain		0.33	0	0	0
**HNL03**			71	M	12		2	2	2	Intermediate ADNC		2.17	0.17	0	1
**HNL04**			57	F	14		0	0	0	Unremarkable Adult Brain		0	0	0	0

Supplementary Table 1: Extended demographics. Diagnostic information is included for the 47 subjects with an "AD continuum" diagnosis and no confounding non-AD tau or frontotemporal lobar degeneration-TDP43 pathology (FTLD-TDP-43) that were included in the pathology-structure correlation analyses. This excludes CNDR01-CNDR07 i.e. cases with Pick's disease (PD), Argyrophilic grain disease (AGD), progressive supranuclear palsy (PSP), corticobasal degeneration (CBD) and FTLD-TDP43. (ADNC: Alzheimer's Disease Neuropathologic Change; LBD: Lewy Body Disease; LATE: Limbic Age-related TDP-43 Encephalopathy; PART: Primary Age-Related Tauopathy; CVD: Cerebrovascular disease; PMI: Post-mortem interval)

**Donor ID**	**3D NFT Burden Map?**	**MTL Subregion Labels?**	**Age**	**Sex**	**PMI (hours)**	**APOE Status**	**Global Pathology Score (NIA/AA Criteria)**			**Neuropathological Diagnosis**		**Contralateral MTL Average Pathology Burden**			
							**A**	**B**	**C**	**Primary**	**Secondary**	**Tau**	**TDP-43**	**a-synuclein**	**AB**
**HNL05**	Y		59	F	3		0	0	0	Unremarkable Adult Brain		0.33	0	0	0
**HNL06**	Y		71	M	10		2	1	2	Low ADNC		0.67	0	0	0
**HNL07**	Y		78	M	8		3	2	2	Intermediate ADNC		1.33	0	0	0
**HNL08**	Y		95	F	9		2	2	3	Intermediate ADNC		1.33	0	0	0.33
**HNL09**	Y		91	F	5		3	2	3	Intermediate ADNC	CAA, LATE	1.83	0.67	0	2
**HNL10**	Y		68	F	6		1	1	0	Low ADNC		1	0	0	0
**HNL11**	Y		74	M	2		1	1	1	Low ADNC		0.33	0	0	2
**HNL12**	Y		90	M	7		2	1	2	Low ADNC	Brainstem predominant Incidental LBD	1	0	0	0.5
**HNL13**	Y		93	F	16		1	1	1	Low ADNC		2	0	0	1
**HNL14**	Y	Y	66	F	9		0	1	0	Unremarkable Adult Brain	Incidental LBD	1	0	0	0
**HNL15**	Y		90	M	6		1	1	0	Low ADNC		0.67	0	0	1
**HNL16**	Y	Y	61	M	16		0	1	0	PART	Mild CBD	1.33	0	0	0
**HNL17**	Y		74	M	3		0	2	0	PART	Mild CAA	1.33	0	0	0
**HNL18**	Y	Y	71	M	10		1	1	0	Low ADNC		0.33	0	0	1
**HNL19**	Y	Y	93	M	11		3	2	3	Intermediate ADNC	CAA	2.17	0	0	3
**HNL20**	Y	Y	78	F	6		1	1	1	Low ADNC	CVD	0.5	0	0	1
**HNL21**			45	M	2		0	1	0	Unremarkable Adult Brain		0.67	0	0	0

Subregion	Sample Size (Low Braak subset/High Braak subset)
SRLM	25 (11/14)
Perforant pathway	25 (11/14)
Dentate Gyrus	25 (11/14)
Cornu Ammonis 1	25 (11/14)
Cornu Ammonus 2	24 (10/14)
Cornu Ammonis 3	24 (10/14)
Subiculum	25 (11/14)
Pre/parasubiculum	24 (10/14)
Entorhinal cortex	25 (11/14)
Brodmann Area 35	25 (11/14)
Brodmann Area 36	24 (10/14)
Area TE	25 (11/14)
Area TF	25 (11/14)
Area TH	24 (11/14)

Supplementary Table 2: Sample sizes for the different MTL subregions in the quantitative NFT burden analyses shown in Figures 2 and 3. In a few cases, certain subregions are missing quantitative NFT burden measures due to exclusion of majority of the tau immunohistochemistry sections in that subregion as a result of poor quality registration or small gaps between tissue blocks.

Subregion	Sample Size (Low Braak subset/High Braak subset)
EC	24 (10/14)
ECL	23 (9/14)
EI	25 (11/14)
ELc	23 (10/13)
ELr	25 (11/14)
EMI	25 (11/14)

Supplementary Table 3: Sample sizes for the different entorhinal cortex subfields in the quantitative NFT burden analyses shown in Figure 6. In a few cases, certain subfields are missing quantitative NFT burden measures due to exclusion of majority of the tau immunohistochemistry sections in that subfield as a result of poor quality registration or small gaps between tissue blocks. (Eo: olfactory; EMI: medial intermediate; EI: intermediate; ELr: lateral rostral; ELc: lateral caudal; EC: caudal; ECL: caudal limiting)

References

[1] D. H. Adler, L. E. Wisse, R. Ittyerah, J. B. Pluta, S. L. Ding, L. Xie, J. Wang, S. Kadivar, J. L. Robinson, T. Schuck, J. Q. Trojanowski, M. Grossman, J. A. Detre, M. A. Elliott, J. B. Toledo, W. Liu, S. Pickup, M. I. Miller, S. R. Das, D. A. Wolk, and P. A. Yushkevich. Characterizing the human hippocampus in aging and Alzheimer's disease using a computational atlas derived from ex vivo MRI and histology. *Proceedings of the National Academy of Sciences of the United States of America*, 115 (16):4252–4257, 2018. ISSN 10916490. doi: 10.1073/pnas.1801093115. URL `http://www.pnas.org/lookup/doi/10.1073/pnas.1801093115`.

[2] J. DeKraker, K. M. Ferko, J. C. Lau, S. Köhler, and A. R. Khan. Unfolding the hippocampus: An intrinsic coordinate system for subfield segmentations and quantitative mapping. *Neuroimage*, 167: 408–418, 2018.

[3] T. Durand, T. Mordan, N. Thome, and M. Cord. Wildcat: Weakly supervised learning of deep convnets for image classification, pointwise localization and segmentation. In *Proceedings of the IEEE conference on computer vision and pattern recognition*, pages 642–651, 2017.

[4] F. Isensee, P. F. Jaeger, S. A. Kohl, J. Petersen, and K. H. Maier-Hein. nnu-net: a self-configuring method for deep learning-based biomedical image segmentation. *Nature methods*, 18(2):203–211, 2021.

[5] W. E. Lorensen and H. E. Cline. Marching cubes: A high resolution 3D surface construction algorithm. In *Proceedings of the 14th Annual Conference on Computer Graphics and Interactive Techniques, SIGGRAPH 1987*, pages 163–169. Association for Computing Machinery, Inc, aug 1987. ISBN 0897912276. doi: 10.1145/37401.37422.

[6] R. Ogniewicz and O. Kübler. Hierarchic Voronoi skeletons. *Pattern Recognition*, 28(3):343–359, mar . ISSN 0031-3203. doi: 10.1016/0031-3203(94)00105-U. URL `https://www.sciencedirect.com/science/article/pii/003132039400105U`.

[7] S. Ravikumar, L. Wisse, Y. Gao, G. Gerig, and P. Yushkevich. Facilitating Manual Segmentation of 3D Datasets using Contour and Intensity Guided Interpolation. In *2019 IEEE 16th International Symposium on Biomedical Imaging (ISBI 2019)*, pages 714–718, 2019. ISBN 9781538636404.

[8] S. Ravikumar, L. E. Wisse, S. Lim, R. Ittyerah, L. Xie, M. L. Bedard, S. R. Das, E. B. Lee, M. D. Tisdall, K. Prabhakaran, et al. Ex vivo mri atlas of the human medial temporal lobe: characterizing neurodegeneration due to tau pathology. *Acta neuropathologica communications*, 9(1):1–14, 2021.

[9] S. Ravikumar, R. Ittyerah, S. Lim, L. Xie, S. Das, P. Khandelwal, L. E. Wisse, M. L. Bedard, J. L. Robinson, T. Schuck, et al. Improved segmentation of deep sulci in cortical gray matter using a deep learning framework incorporating laplace's equation. In *International Conference on Information Processing in Medical Imaging*, pages 692–704. Springer, 2023.

[10] N. J. Tustison, B. B. Avants, P. A. Cook, Y. Yuanjie Zheng, A. Egan, P. A. Yushkevich, and J. C. Gee. N4ITK: Improved N3 Bias Correction. *IEEE Transactions on Medical Imaging*, 29(6):1310–1320, jun 2010. ISSN 0278-0062. doi: 10.1109/TMI.2010.2046908. URL `http://www.ncbi.nlm.nih.gov/pubmed/20378467http://www.pubmedcentral.nih.gov/articlerender.fcgi?artid=PMC3071855http://ieeexplore.ieee.org/document/5445030/`.

[11] P. A. Yushkevich, M. M. López, M. M. I. d. O. Martin, R. Ittyerah, S. Lim, S. Ravikumar, M. L. Bedard, S. Pickup, W. Liu, J. Wang, L. Y. Hung, J. Lasserve, N. Vergnet, L. Xie, M. Dong, S. Cui, L. McCollum, J. L. Robinson, T. Schuck, R. de Flores, M. Grossman, M. D. Tisdall, K. Prabhakaran, G. Mizsei, S. R. Das, E. Artacho-Pérula, M. d. M. A. Jiménez, M. P. M. Rabal, F. J. M. Romero, S. C. Sánchez, J. C. D. González, C. de la Rosa-Prieto, M. C. Parada, E. B. Lee, J. Q. Trojanowski, D. T. Ohm, L. E. M. Wisse, D. A. Wolk, D. J. Irwin, and R. Insausti. Three-dimensional mapping of neurofibrillary tangle burden in the human medial temporal lobe. *Brain*, 139(4):16–17, 2021. doi: 10.1093/BRAIN/AWAB262. URL `https://academic.oup.com/brain/advance-article/doi/10.1093/brain/awab262/6321227`.
