# Supplementary material for: Postmortem imaging reveals patterns of medial temporal lobe vulnerability to tau pathology in Alzheimer’s disease
Source: Nat Commun. 2024 Jun 5;15:4803. doi: 10.1038/s41467-024-49205-0 (PMC11153494; doi:10.1038/s41467-024-49205-0)
Supplement: Supplementary file 3 — Description of Additional Supplementary Files [file 41467_2024_49205_MOESM3_ESM.pdf]

Description of Additional Supplementary Files

File Name: Supplementary Movie 1
Description: 3-D volume renderings of the average NFT burden maps reconstructed in the space of the ex vivo MRI atlas of the medial temporal lobe. Average maps are computed separately for specimens with a low B score (B0 or B1, which corresponds to Braak stages 0-II; n = 11) and high B score (B2 or B3, which corresponds to Braak stages III-VI; n=14).
