# Supplementary material for: Postmortem imaging reveals patterns of medial temporal lobe vulnerability to tau pathology in Alzheimer’s disease
Source: Nat Commun. 2024 Jun 5;15:4803. doi: 10.1038/s41467-024-49205-0 (PMC11153494; doi:10.1038/s41467-024-49205-0)
Supplement: Supplementary file 5 — Reporting Summary [file 41467_2024_49205_MOESM5_ESM.pdf]

nature portfolio

Corresponding author(s): Sadhana Ravikumar

Last updated by author(s): Mar 14, 2024

Reporting Summary

Nature Portfolio wishes to improve the reproducibility of the work that we publish. This form provides structure for consistency and transparency in reporting. For further information on Nature Portfolio policies, see our Editorial Policies and the Editorial Policy Checklist.

Statistics

For all statistical analyses, confirm that the following items are present in the figure legend, table legend, main text, or Methods section.

n/a	Confirmed	
☐	☒	The exact sample size (n) for each experimental group/condition, given as a discrete number and unit of measurement
☐	☒	A statement on whether measurements were taken from distinct samples or whether the same sample was measured repeatedly
☐	☒	The statistical test(s) used AND whether they are one- or two-sided<br>*Only common tests should be described solely by name; describe more complex techniques in the Methods section.*
☐	☒	A description of all covariates tested
☐	☒	A description of any assumptions or corrections, such as tests of normality and adjustment for multiple comparisons
☐	☒	A full description of the statistical parameters including central tendency (e.g. means) or other basic estimates (e.g. regression coefficient) AND variation (e.g. standard deviation) or associated estimates of uncertainty (e.g. confidence intervals)
☐	☒	For null hypothesis testing, the test statistic (e.g. F, t, r) with confidence intervals, effect sizes, degrees of freedom and P value noted<br>*Give P values as exact values whenever suitable.*
☒	☐	For Bayesian analysis, information on the choice of priors and Markov chain Monte Carlo settings
☒	☐	For hierarchical and complex designs, identification of the appropriate level for tests and full reporting of outcomes
☐	☒	Estimates of effect sizes (e.g. Cohen's d, Pearson's r), indicating how they were calculated

Our web collection on statistics for biologists contains articles on many of the points above.

Software and code

Policy information about availability of computer code

Data collection	No software was used for data collection.
Data analysis	Pre-processing of MRI scans with open-source tool convert3d (v1.1.0 - https://github.com/pyushkevich/c3d) and ANTs (v2.3.5)<br>Semi-automated and automated segmentation of the ex vivo MRI scans was done with ITK-SNAP (v4.0.1) and a custom implementation of nnU-Net based on nnU-Net v1 respectively. A docker container for implementing the custom deep-learning based ex vivo cortical segmentation is publicly available (https://github.com/sadhana-r/exvivoMTLsegmentation_docker).<br>The group-wise registration pipeline was implemented with open-source tools, greedy (v1.2.0 - https://github.com/pyushkevich/greedy) and cmrep (v1.0.0 - https://github.com/pyushkevich/cmrep). Our code for atlas construction is publicly available: https://github.com/sadhana-r/exvivo_tau_atlas_scripts (DOI: 10.5281/zenodo.11123538.).<br>Code for 3D reconstruction of histology sections and registration to MRI is publicly available: https://github.com/pyushkevich/tau_recon_scripts.<br>Mesh-based statistical analysis with open-source tool meshglm, implemented in cmrep, and ROI-based analysis with R (v4.3.0).<br>Imaging data visualization with ITK-SNAP (v4.0.1) and Paraview (v5.9).

For manuscripts utilizing custom algorithms or software that are central to the research but not yet described in published literature, software must be made available to editors and reviewers. We strongly encourage code deposition in a community repository (e.g. GitHub). See the Nature Portfolio guidelines for submitting code & software for further information.

Data

Policy information about availability of data

All manuscripts must include a data availability statement. This statement should provide the following information, where applicable:

- Accession codes, unique identifiers, or web links for publicly available datasets
- A description of any restrictions on data availability
- For clinical datasets or third party data, please ensure that the statement adheres to our policy

The anonymized raw and processed data including the subject MRI scans, and subject-level and group-level histology-based segmentations, and quantitative NFT burden maps generated in this study have been deposited in the OpenNeuro database under accession code ds004767 (https://doi.org/10.18112/openneuro.ds004767.v1.0.0). Detailed demographics, neuropathological diagnosis and semi-quantitative neuropathology ratings for each donor are provided in the Supplementary Information file. The medial temporal lobe subregion-level quantitative tau burden measures and mean thickness measurements used in this study are provided in the Source Data.

Research involving human participants, their data, or biological material

Policy information about studies with human participants or human data. See also policy information about sex, gender (identity/presentation), and sexual orientation and race, ethnicity and racism.

Reporting on sex and gender	Table 1 and Supplementary Table 1 present summary and individual-level demographic information, including sex, for the brain donor cohorts used in the different analyses. We also report the results of our analyses with sex included as a co-variate.
Reporting on race, ethnicity, or other socially relevant groupings	Race/ethnicity were not used as variables in the analysis.
Population characteristics	Table 1 and Supplementary Table 1 present summary and individual-level donor characteristics and in the analyses presented in Figures 4 and 5, we included the age at death for each donor as a covariate. We also report the results of our analyses with correction for sex (Figure 5, Supplementary Figure 4).
Recruitment	Donors from the Center for Neurodegenerative Disease Research were participants in in vivo ageing and dementia research and included patients from the Penn Frontotemporal Degeneration Center and the Penn Alzheimer's Disease Research Center.
Ethics oversight	The research was deemed exempt by the University of Pennsylvania Institutional Review Board, as it involves postmortem human brain specimens. Human brain specimens from the University of Pennsylvania and Human Neuroanatomy Lab at University of Castilla-La Mancha (UCLM) were obtained in accordance with the local laws and regulations and the Ethical Committee of UCLM respectively. Consent to autopsy and research use of brain tissue was provided by the next of kin in all brain donations.

Note that full information on the approval of the study protocol must also be provided in the manuscript.

Field-specific reporting

Please select the one below that is the best fit for your research. If you are not sure, read the appropriate sections before making your selection.

[x] Life sciences [ ] Behavioural & social sciences [ ] Ecological, evolutionary & environmental sciences

For a reference copy of the document with all sections, see nature.com/documents/nr-reporting-summary-flat.pdf

Life sciences study design

All studies must disclose on these points even when the disclosure is negative.

Sample size	For this cross-sectional study, a priori sample size calculations for power analysis were not performed. The ex vivo atlas was constructed from 55 temporal lobe specimens. Structure-pathology correlations using semi-quantitative neuropathology measurements were performed in a subset of 47 specimens, and quantitative 3D tau burden maps were analyzed in 25 specimens. Considering the time intensive nature of postmortem data collection and histological analysis, the presented dataset is considerably large for a postmortem dataset.
Data exclusions	For this study, we were interested in characterizing neurodegeneration in patients with a primary diagnosis of Alzheimer's disease pathology. Structure-pathology correlations were examined in cases with an "AD continuum" diagnosis and no confounding non-AD tau (i.e., Pick's disease, progressive supranuclear palsy and corticobasal degeneration) or FTLD-TDP43 pathology. AD continuum diagnoses included cases with a neuropathological diagnosis of 'unremarkable brain', primary age-related tauopathy and AD neuropathologic change, and co-pathologies such as Lewy body dementia, cerebrovascular disease, multiple system atrophy, limbic age-related TDP-43 encephalopathy, hippocampal sclerosis and cerebral amyloid angiopathy. Additionally, cases were excluded if the specimen's MRI scan had too many artifacts, due to tissue tearing and air bubbles, and insufficient coverage of the medial temporal lobe to allow for reliable group-wise registration and thickness estimation.

Replication	The presented ex vivo atlas and 3D reconstructed NFT burden maps were generated by re-implementing and improving previously developed pipelines using a much larger postmortem dataset, thus highlighting the reproducibility of the developed methods across different specimens and datasets.
Randomization	Randomization was not used in this study
Blinding	During the data collection and processing, the personnel performing these steps were not aware of the donors neuropathological diagnosis.

Reporting for specific materials, systems and methods

We require information from authors about some types of materials, experimental systems and methods used in many studies. Here, indicate whether each material, system or method listed is relevant to your study. If you are not sure if a list item applies to your research, read the appropriate section before selecting a response.

Materials & experimental systems

n/a	Involved in the study
☐	☒ Antibodies
☒	☐ Eukaryotic cell lines
☒	☐ Palaeontology and archaeology
☒	☐ Animals and other organisms
☒	☐ Clinical data
☒	☐ Dual use research of concern
☒	☐ Plants

Methods

n/a	Involved in the study
☒	☐ ChIP-seq
☒	☐ Flow cytometry
☐	☒ MRI-based neuroimaging

Antibodies

Antibodies used	For the routine semi-quantitative neuropathology examinations, the following antibodies were used:<br>- PHF-1 (mAb, 1:1000, a gift from Dr. Peter Davies) to detect phosphorylated tau deposits (phosphorylated at Ser396 and Ser404). Host species: mouse monoclonal, IgG1<br>- NAB228 (monoclonal antibody [mAb], 1:8000, generated in the CNDR) to detect amyloid-β deposits<br>- pS409/410 (mAb, 1:500, a gift from Dr. Manuela Neumann and Dr. E. Kremmer) to detect phosphorylated TDP-43 deposits<br>- Syn303 (mAb, 1:16,000, generated in the CNDR) to detect the presence of the pathological conformation of α-synuclein<br><br>For the quantitative serial histological analysis, tissue sections were stained with AT8 (Host species: mouse monoclonal, IgG1), a human phosphorylated tau antibody immunohistochemistry stain, and counterstained for Nissl.
Validation	We always included a positive control when we used these antibodies for immunohistochemistry staining.

Plants

Seed stocks	*Report on the source of all seed stocks or other plant material used. If applicable, state the seed stock centre and catalogue number. If plant specimens were collected from the field, describe the collection location, date and sampling procedures.*
Novel plant genotypes	*Describe the methods by which all novel plant genotypes were produced. This includes those generated by transgenic approaches, gene editing, chemical/radiation-based mutagenesis and hybridization. For transgenic lines, describe the transformation method, the number of independent lines analyzed and the generation upon which experiments were performed. For gene-edited lines, describe the editor used, the endogenous sequence targeted for editing, the targeting guide RNA sequence (if applicable) and how the editor was applied.*
Authentication	*Describe any authentication procedures for each seed stock used or novel genotype generated. Describe any experiments used to assess the effect of a mutation and, where applicable, how potential secondary effects (e.g. second site T-DNA insertions, mosiacism, off-target gene editing) were examined.*

Magnetic resonance imaging

Experimental design

Design type	Postmortem structural MRI of temporal lobe specimens was utilized to construct an anatomical atlas and measure thickness of the cortex.
Design specifications	Temporal lobe specimens were scanned in a cylindrical container containing an MRI neutral industrial lubricant. The specimens were imaged with a custom-made 70 mm coil, using an acrylic holder with a 49.8 mm inner diameter and a long (80 mm) z-field in a Varian 9.4 T animal scanner.

Behavioral performance measures | Not applicable

Acquisition

Imaging type(s)	structural MRI
Field strength	9.4 T postmortem
Sequence & imaging parameters	MRI scans were obtained using a multi-slice spin echo sequence, with an isotropic resolution of 0.2mm x 0.2mm x 0.2mm. Sequence parameters varied slightly between specimens, with typical values being a repetition time of 9330 ms and an echo time of 23 ms.
Area of acquisition	postmortem human temporal lobe specimens

Diffusion MRI ☐ Used ☒ Not used

Preprocessing

Preprocessing software	Images were corrected for bias field non-uniformity using the N4ITK algorithm in ANTs. Due to an error in the scanner gradient calibration, as part of the pre-processing, all the scans had to be linearly scaled (6% in x, 3% in y, 11% in z) to correct for differences between the scanner coordinate frame and physical coordinate frame. These linear scaling factors were derived using a 3D printed phantom. More details on this are provided in Adler et al. PNAS (2018).
Normalization	Using the open-source tool, convert3d, MRI scans were normalized to a common intensity range by clipping the intensities below the 0.1 and above the 99.9 percentile, and scaling the intensity range to [0,1000].
Normalization template	A custom template was constructed using the methods described in Section 4.2 "Construction of a computational ex vivo MRI atlas", with additional details provided in Ravikumar et al. Acta Neuropathologica Communications (2021). The open-source tools "greedy" and "cmrep" were used to perform registration between specimens.
Noise and artifact removal	Prior to scanning, special precaution was taken to eliminate air bubbles trapped in both the brain and container by gently agitating the specimen by hand while submerged in the MRI-neutral lubricant. N4 bias field correction was performed using the ANTs software.
Volume censoring	Not applicable

Statistical modeling & inference

Model type and settings	Linear correlations and Spearman rank correlations were performed to relate thickness measured from MRI with histology-based neuropathology measurements.
Effect(s) tested	See the Methods section on "Group-level statistical analysis". In the pointwise thickness analyses, we tested the effect of semi-quantitative, contralateral ratings of tau, TDP-43 and alpha-synuclein pathologies on thickness. For the ROI-based analyses, we tested the effect of quantitative neurofibrillary tangle measures, derived ipsilaterally, on thickness.

Specify type of analysis: ☐ Whole brain ☐ ROI-based ☒ Both

Anatomical location(s)	Our analyses include both point-wise and ROI-based analyses. For each specimen, the medial temporal lobe was divided in to 26 anatomical regions-of-interest (ROI) based on a consensus histology-based MTL subregion segmentation in the space of the ex vivo atlas, derived from serial histology imaging in 17 specimens. For each of the 17 specimens, borders between subregions were first traced on serial Nissl digital histology images based on cytoarchitectural features. These borders were then mapped to 9.4T MRI space and used to trace 3-D segmentations of the subregions. Following atlas construction, the 17 completed histology-based MTL subregion segmentations were mapped to the space of the MRI atlas using the deformable transformations generated by the group-wise registration pipeline, and a consensus labeling of the MRI atlas was obtained by application of voxel-wise majority voting among the 17 segmentations with slight regularization by a Markov Random Field prior.
Statistic type for inference (See Eklund et al. 2016)	Cluster-wise and ROI-based analysis. For cluster-level analysis, clusters were defined based on an empirical threshold (uncorrected $p < 0.01$) and permutation testing with the Freedman & Lane method (1000 iterations).
Correction	See Methods section on "Group-level statistical analysis". For the point-wise mesh based analysis, correction for multiple comparisons was performed using cluster-level family wise error rate correction. For the ROI-level analysis in the smaller subset of 25 specimens, correction for multiple comparisons was not applied.

Models & analysis

n/a	Involved in the study
☒	☐ Functional and/or effective connectivity
☒	☐ Graph analysis
☐	☒ Multivariate modeling or predictive analysis

Multivariate modeling and predictive analysis

To model the relationship between cortical thickness, measured using MRI, and the different neurodegenerative pathologies, we included the neuropathology measurement of interest as the independent variable, thickness as the dependent variable, and age, sex and co-pathology measurements as nuisance covariates (Figure 2).
